# Supplementary material for: Mitochondrial Bol1 and Bol3 function as assembly factors for specific iron-sulfur proteins
Source: eLife. 2016 Aug 17;5:e16673. doi: 10.7554/eLife.16673 (PMC5014550; doi:10.7554/eLife.16673)
Supplement: Supplementary file 1. — (A) Yeast strains used in this study. Gene disruptions and promoter exchanges were generated by PCR-based gene replacement and verified by PCR as described previously (Gueldener et al., 2002; Mühlenhoff et al., 2002). Yeast cells were transformed by the lithium acetate method (Gietz and Woods, 2002). In some cells, the TRP1 gene was disrupted by a natNT2 cassette (Janke et al., 2004) in order utilize plasmids with the TPR1 marker. (B) Plasmid constructs used in this study. The plasmids were constructed inserting the indicated genes into vector. The amino acid residues of the encoded proteins and the hexa-histidinyl tag (6xHis) are indicated. DOI: http://dx.doi.org/10.7554/eLife.16673.026 [file elife-16673-supp1.docx]

**Supplementary Information**

**Mitochondrial Bol1 and Bol3 function**

**as assembly factors for specific iron-sulfur proteins**

Marta A. Uzarska, Veronica Nasta, Benjamin D. Weiler, Farah Spantgar, Simone Ciofi-Baffoni, Maria Rosaria Saviello, Leonardo Gonnelli, Ulrich Mühlenhoff, Lucia Banci, and Roland Lill

**Supplementary File 1A: Yeast Strains Used in This Study**

| **Strain** | **Genotype** | **Method of Generation** | **Source/Reference** |
| --- | --- | --- | --- |
| W303-1A | *MATa; ura3-1; ade2-1; trp1-1; his3-11,15; leu2-3,112* |  | (Mortimer and Johnston, 1986) |
| BY4742 | *MATα; his3Δ1; leu2Δ0; met15Δ0; ura3Δ0* | obtained from Euroscarf | (Brachmann et al., 1998) |
| *bol1*Δ | BY4739*, yal45c:: KanMX4* | obtained from Euroscarf | EUROSCARF |
| *bol2*Δ | BY4742*, ygl220w:: KanMX4* | obtained from Euroscarf | EUROSCARF |
| *bol3*Δ | BY4742*, yal46c:: KanMX4* | obtained from Euroscarf | EUROSCARF |
| *bol12*Δ | *bol1*Δ*, ygl220w:: HIS3* | PCR Fragment (pFA6a-HIS3) (Euroscarf) | this work |
| *bol13*Δ | *yal44w,yal46c::LEU2;*  *leu2::HIS3* | PCR Fragment (pUG73) PCR Fragment (pFA6a-HIS3) (Euroscarf) | this work |
| *bol123*Δ | *bol13*Δ*, ygl220w:: KanMX4* | obtained from Euroscarf | this work |
| *bol23*Δ | *bol3*Δ*, ygl220w:: HIS3* | obtained from Euroscarf | this work |
| *grx5Δ* | W303-1A, *grx5::KanMX4* | PCR fragment (pFA6a-KanMX4) | (Rodriguez-Manzaneque et al., 1999) |
| *lip5*Δ | BY4742*, lip5:: KanMX4* | obtained from Euroscarf | EUROSCARF |
| *nfu1Δ* | BY4742 *nfu1:: KanMX4* | obtained from Euroscarf | EUROSCARF |
| *nfu1*Δ*bol1*Δ | *bol1*Δ*, nfu1::natNT2* | PCR fragment (pFA6a–natNT2) (Janke et al., 2004) | this work |
| *nfu1*Δ*bol3*Δ | *bol3*Δ*, nfu1::natNT2* | PCR fragment (pFA6a–natNT2) (Janke et al., 2004) | this work |
| *nfu1*Δ*bol13*Δ | *bol13*Δ*, nfu1::natNT2* | PCR fragment (pFA6a–natNT2) (Janke et al., 2004) | this work |

Gene disruptions and promoter exchanges were generated by PCR-based gene replacement and verified by PCR as described previously (Gueldener et al., 2002; Mühlenhoff et al., 2002)Yeast cells were transformed by the lithium acetate method (Gietz and Woods, 2002). In some cells, the *TRP1* gene was disrupted by a *natNT2* cassette (Janke et al., 2004) in order utilize plasmids with the *TPR1* marker.

**Supplementary File 1B. Plasmid Constructs Used in This Study**

| **plasmid** | **ORF** | **backbone** | **Source/Reference** |
| --- | --- | --- | --- |
| p424-*BOL1-Myc* | *BOL1,* C-terminal Myc | p424-*MET25* (Mumberg et al., 1995) | this work |
| p425-*BOL3-HA* | *BOL3,* C-terminal HA | p425-*TDH3* (Mumberg et al., 1995) | this work |
| p414-*BOL1* | *BOL1* | p414-*MET25* (Mumberg et al., 1995) | this work |
| p414-*BOL2* | *BOL2,* C-terminal HA | p414-*MET25* | this work |
| p414-*BOL3* | *BOL3* | p414-*MET25* | this work |
| p414-*BOL13* | *BOL13,* own promoter replacing *MET25* | p414-*MET25* | this work |
| p414-*SDH2-Myc* | *SDH2;* C-terminal Myc | p414-*MET25* | Gift of D.R.Winge |
| p416-*SDH1* | *SDH1* | p416-*MET25* | Gift of D.R.Winge |
| p426-*HiPIP-Myc* | Pre-F1*β *(1-40)-[4Fe/4S]-*HIPIP*; (*C. vinosum);* C-terminal Myc | p426-*TDH3* (Mumberg et al., 1995) | (Mühlenhoff et al., 2011) |
| p426-*RLI1-HA* | *RLI1;* C-terminal HA; own promoter replacing *TDH3* promoter | p426-*TDH3* | (Kispal et al., 2005) |
| p*FET3*-*GFP* | *GFP; FET3* promoter replacing *MET25* promoter | p416-*MET25* | (Hausmann et al., 2008) |
| p*FIT3*-*luc2* | *GFP; FIT3* promoter replacing *MET25* promoter | p416-*MET25* | this work |
| p426-*FDX2-HA* | *FDX2* (*Homo sapiens);* C-terminal HA | p426-*TDH3* | (Sheftel et al., 2010) |
| p424-*GRX5* | *GRX5* | p424-*TDH3* | (Uzarska et al., 2013) |
| p424-*SpGRX5-Myc* | *GRX5 (Schizosaccharomyces pombe);* C-terminal Myc | p424-*TDH3* | (Uzarska et al., 2013) |
| p416-*NFU1* | *NFU1* | p416-*MET25* | (Navarro-Sastre et al., 2011) |
| pETDuet-1-*GLRX5* | truncated *Homo sapiens GLRX5* (32-157)*;* N-terminal 6xHis | pETDuet-1 (Novagen) | this work |
| pETDuet-1-*BOLA1* | truncated *Homo sapiens BOLA1* (30-137)*;* N-terminal 6xHis | pETDuet-1 | this work |
| pETDuet-1-*BOLA3* | truncated *Homo sapiens BOLA3* (25-107)*;* N-terminal 6xHis | pETDuet-1 | this work |
| pETDuet-1-*NFU1* | truncated *Homo sapiens NFU1* (57-254)*;* N-terminal 6xHis | pETDuet-1 | this work |

The plasmids were constructed inserting the indicated genes into vector. The amino acid residues of the encoded proteins and the hexa-histidinyl tag (6xHis) are indicated.

**References:**

Brachmann, C.B., Davies, A., Cost, G.J., Caputo, E., Li, J., Hieter, P., and Boeke, J.D. (1998). Designer deletion strains derived from Saccharomyces cerevisiae S288C: a useful set of strains and plasmids for PCR-mediated gene disruption and other applications. Yeast *14*, 115-132.

Gietz, R.D., and Woods, R.A. (2002). Transformation of yeast by lithium acetate/single-stranded carrier DNA/polyethylene glycol method. Methods Enzymol *350*, 87-96.

Gueldener, U., Heinisch, J., Koehler, G.J., Voss, D., and Hegemann, J.H. (2002). A second set of loxP marker cassettes for Cre-mediated multiple gene knockouts in budding yeast. Nucleic Acids Res *30*, e23.

Hausmann, A., Samans, B., Lill, R., and Muhlenhoff, U. (2008). Cellular and Mitochondrial Remodeling upon Defects in Iron-Sulfur Protein Biogenesis. J Biol Chem *283*, 8318-8330.

Janke, C., Magiera, M.M., Rathfelder, N., Taxis, C., Reber, S., Maekawa, H., Moreno-Borchart, A., Doenges, G., Schwob, E., Schiebel, E., et al. (2004). A versatile toolbox for PCR-based tagging of yeast genes: new fluorescent proteins, more markers and promoter substitution cassettes. Yeast *21*, 947-962.

Kispal, G., Sipos, K., Lange, H., Fekete, Z., Bedekovics, T., Janaky, T., Bassler, J., Aguilar Netz, D.J., Balk, J., Rotte, C., et al. (2005). Biogenesis of cytosolic ribosomes requires the essential iron-sulphur protein Rli1p and mitochondria. EMBO J. *24*, 589-598.

Mortimer, R.K., and Johnston, J.R. (1986). Genealogy of principal strains of the yeast genetic stock center. Genetics *113*, 35-43.

Mühlenhoff, U., Richhardt, N., Ristow, M., Kispal, G., and Lill, R. (2002). The yeast frataxin homologue Yfh1p plays a specific role in the maturation of cellular Fe/S proteins. Hum. Mol. Genet. *11*, 2025-2036.

Mühlenhoff, U., Richter, N., Pines, O., Pierik, A.J., and Lill, R. (2011). Specialized function of yeast Isa1 and Isa2 proteins in the maturation of mitochondrial [4Fe-4S] proteins. J Biol Chem *286*, 41205-41216.

Mumberg, D., Müller, R., and Funk, M. (1995). Yeast vectors for controlled expression of heterologous proteins in different genetic backgrounds. Gene *156*, 119-122.

Navarro-Sastre, A., Tort, F., Stehling, O., Uzarska, M.A., Arranz, J.A., Del Toro, M., Labayru, M.T., Landa, J., Font, A., Garcia-Villoria, J., et al. (2011). A fatal mitochondrial disease is associated with defective NFU1 function in the maturation of a subset of mitochondrial Fe-S proteins. Am J Hum Genet *89*, 656-667.

Rodriguez-Manzaneque, M.T., Ros, J., Cabiscol, E., Sorribas, A., and Herrero, E. (1999). Grx5 glutaredoxin plays a central role in protection against protein oxidative damage in Saccharomyces cerevisiae. Mol Cell Biol *19*, 8180-8190.

Sheftel, A.D., Stehling, O., Pierik, A.J., Elsasser, H.P., Muhlenhoff, U., Webert, H., Hobler, A., Hannemann, F., Bernhardt, R., and Lill, R. (2010). Humans possess two mitochondrial ferredoxins, Fdx1 and Fdx2, with distinct roles in steroidogenesis, heme, and Fe/S cluster biosynthesis. Proc Natl Acad Sci U S A *107*, 11775-11780.

Uzarska, M.A., Dutkiewicz, R., Freibert, S.A., Lill, R., and Muhlenhoff, U. (2013). The mitochondrial Hsp70 chaperone Ssq1 facilitates Fe/S cluster transfer from Isu1 to Grx5 by complex formation. Mol Biol Cell *24*, 1830-1841.
